# LONGITUDINAL NORMS OF COGNITIVE TESTS IN THE ENGLISH LONGITUDINAL STUDY OF AGING

**DOI:** 10.1093/geroni/igad104.2949

**Published:** 2023-12-21

**Authors:** Alejandra Marroig, Franklin Massa, Graciela Muniz-Terrera

**Affiliations:** Universidad de la Republica Uruguay, Montevideo, Montevideo, Uruguay; Universidad de la República, Montevideo, Montevideo, Uruguay; Ohio University, Athens, Ohio, United States

## Abstract

Longitudinal research on cognitive change has mostly been focused on mean change in cognition and its associated factors. In this paper, we aim to assess the role of socio-demographic characteristics on different percentiles of the distribution of cognitive trajectories considering sample attrition due to death and intermittent missing data. We used data from 9 waves of the English Longitudinal Study of Ageing (ELSA) covering the period 2002-2019. We consider the numeracy and literacy recall to assess cognition of older adults and applied weighted Generalized Estimating Equations to estimate the 10th, 50th (median), and 90th percentiles of the cognitive tests trajectories. We adjust for sample attrition due to death and assess the role of sex and education on the cognitive trajectories. The results show differences in the cognitive trajectories across the percentiles. There is a steeper cognitive decline for individuals with poorer cognitive condition (p< 0.001). Sociodemographic characteristics are associated with cognitive decline but there is heterogeneity across cognitive trajectory percentiles. Education is protective against cognitive decline but it is more effective for individuals with lower cognitive impairment (p< 0.001). In this approach we advance the understanding of cognitive trajectories of older adults, showing differences across percentiles. We address the process of missing information and the results show new insights. Thus, this may improve the practice and design of interventions aimed at cognitive decline of older adults. Our results provide important insights into longitudinal norms of cognitive decline in a population-representative of older adults.

